# The P granule antibody KT3 recognizes epitopes in both PGL-1 and PGL-3

**DOI:** 10.17912/micropub.biology.001137

**Published:** 2024-02-16

**Authors:** Brennan M Danlasky, Mario Martinez, Madeline Cassani, Devavrat Bodas, Andrea Putnam, Geraldine Seydoux

**Affiliations:** 1 Molecular Biology and Genetics, Howard Hughes Medical Institute, Johns Hopkins University School of Medicine; 2 Molecular Biology and Genetics, University of Wisconsin–Madison, Howard Hughes Medical Institute, Johns Hopkins University School of Medicine

## Abstract

The KT3 antibody is a commercially available antibody that recognizes the P granule protein PGL-3 (Takeda et al., 2008). Using immunostaining and western blotting of purified peptide fragments, we show that KT3 recognizes both PGL-3 and its paralog PGL-1 , likely through a shared epitope in the intrinsically disordered region.

**Figure 1. The P granule antibody KT3 recognizes epitopes in both PGL-1 and PGL-3:**
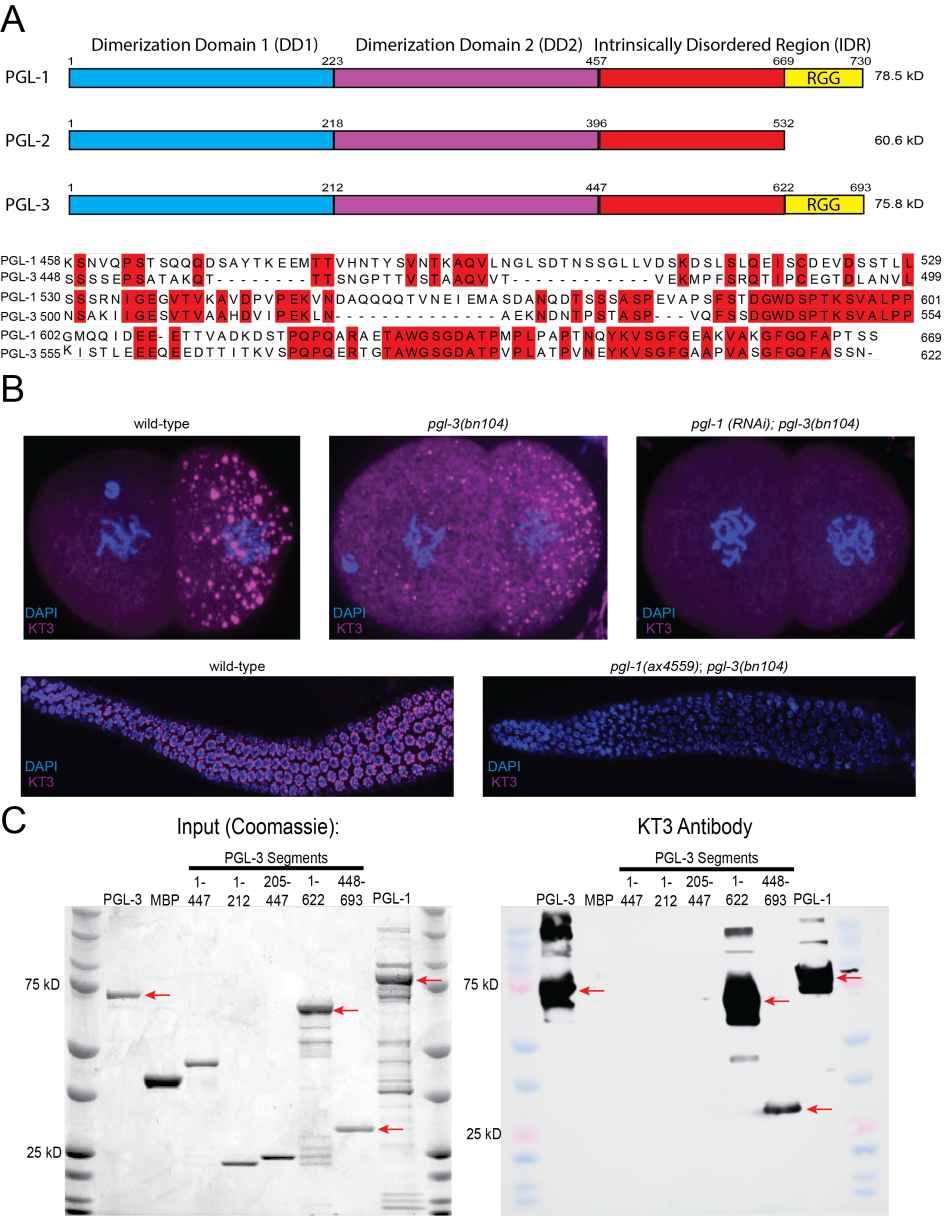
A. PGL proteins Diagram showing different domains in PGL-1 , PGL-2 and PGL-3 and MUSCLE alignment of the PGL-1 and PGL-3 intrinsically disordered regions (not including the RGG repeats). Regions of homology between PGL-1 and PGL-3 are highlighted in red and may correspond to regions recognized by KT3. The PGL-2 IDR did not show significant stretches of homology and thus was excluded from this analysis. B: Immunostaining analyses 2-cell stage embryos (top row) and dissected germlines (bottom row) of indicated genotype probed with KT3 (purple). C: Western analyses Coomassie-stained gel (left panel) and western blot of same probed with KT3 antibody (right panel). Recombinant proteins were purified as fusions with maltose binding protein (MBP) and cleaved to release MBP and untagged PGL. Arrows point to bands recognized by the KT3 antibody. Bands of higher-than-expected molecular weight likely correspond to uncleaved MBP fusions. PGL-3 is full length, MBP is maltose binding protein. The next five lanes are PGL-3 segments as indicated (Refer to diagram A for amino acid coordinates). PGL-1 is full length PGL-1 .

## Description

The KT3 antibody and other antibodies obtained via antigen subtraction are widely used reagents for studying *C. elegans* (Takeda et al. 2008, Hanazawa et al. 2011, Schmidt et al., 2021) *.* Antigen subtraction is a two round hybridoma production and screening method designed to raise monoclonal antibodies against random, relatively low abundance proteins present in a tissue lysate. The KT3 antibody was originally characterized (Takeda et al., 2008) as an antibody against PGL-3 , a 75kD P granule protein (Kawasaki et al., 2004) . In immunostaining experiments in embryos, KT3 recognizes P granules and an unidentified ( *pgl-3* independent) epitope in muscle (Takeda et al., 2008) .

*C. elegans* has three PGL paralogs, each with two conserved N-terminal dimerization domains and a less conserved intrinsically disordered region (IDR) ( Fig. 1A, Kawazaki et al ., 2004). The PGL-1 and PGL-3 IDRs share some homology with each other, but not with the PGL-2 IDR. In immunostaining of embryos, we noticed that KT3 recognizes P granules in both wild-type and *pgl-3 ( bn104 )* knockout embryos *,* suggesting that KT3 might recognize another P granule epitope besides PGL-3 ( Fig. 1B ). Consistent with this view, no P granule staining was observed in *pgl-3 ( bn104 ); pgl-1 (RNAi)* embryos and adult *pgl-3 ( bn104 ); pgl-1 ( ax4559 )* germlines, suggesting that KT3 also recognizes PGL-1 , but not PGL-2 .

To further define KT3 specificity, we tested KT3 against recombinant PGL-1 , PGL-3 , and PGL-3 segments partially purified from *E. coli* ( Fig. 1C ). The KT3 antibody recognized full length PGL-1 and PGL-3 , as well as the PGL-3 IDR and a truncated PGL-3 lacking the RGG domain. KT3 did not recognize PGL-3 segments lacking the IDR.

We conclude that KT3 recognizes P granule proteins PGL-1 and PGL-3 , likely through a shared epitope in the IDR.

## Methods

**CRISPR generation of PGL-1 deletion**

JH4230 *pgl-1 ( ax4559 ); pgl-3 ( bn104 )* was generated via CRISPR/CAS9 genome editing as described in Paix et al., 2017.

**Protein purification**

MBP::6XHis::TEV:: PGL-3 was expressed in Rosetta (DE3) cells in terrific broth + ampicillin (100 μg/mL) to an OD600 of ~1.0 at 37°C and induced with 1 mM isopropyl β-D-1-thiogalactopyranoside at 16° C for 16 hr. Cells were resuspended in Buffer A (25 mM HEPES pH 7.4, 500 mM NaCl, 0.4 M L-Arginine, 20% (vol/vol) glycerol, 1 mM DTT) with added protease inhibitors, 5 μg/mL RNase A (USB), 0.25 U/μL RNase I (Ambion), lysed by sonication, spun at 13,000 rpm for 15 min. Lysates were filtered and incubated with amylose resin (New England Biolabs). The resin was washed 3X with Buffer B (25 mM HEPES pH 7.4, 20% (vol/vol) glycerol, 1 mM DTT, 500 mM NaCl). Protein was eluted in Buffer B + 20 mM Maltose. 6XHis::TEV::5XArg tag protease (expression plasmid obtained from addgene (PRK793) and purified as described (Tropea, Cherry, and Waugh 2009) was added at a ratio of ~ 1:5 mg TEV:PGL and incubated O/N at 16° C. After TEV cleavage MBP::6XHis and 6XHis::TEV::5XArg protease were removed by passing the cleavage reaction through a 5 mL HisTRAP column (GE Healthcare). The flowthrough containing cleaved, untagged PGL-3 was diluted to ~150 mM NaCl with Buffer B0 (containing 0 mM NaCl) and immediately loaded onto a HeparinTrap column (GE Healthcare). Proteins were eluted with a gradient of 150 to 1000 mM NaCl. PGL-3 mutants lacking the c-terminal RGG domain did not bind to the heparin column and were collected from the flowthrough and applied to a 5 mL HiTrap Q column (GE Healthcare). Proteins were eluted with a gradient of 150 to 1000 mM NaCl. Protein containing fractions were concentrated and flash-frozen in small aliquots in liquid nitrogen and stored at −80°C. Protocol adapted from Folkmann et al. 2021 and Putnam et al., 2019.

**RNAi**

L4 larvae were placed on IPTG-induced lawns of HT115 bacteria bearing L4440-based plasmid for 24 hours at 25°C followed by fixation, and then shifted to 20°C for one hour to avoid temperature dependent changes on P granules. The RNAi clone for *pgl-1* came from the genomic RNAi feeding library (Medical Research Council Gene Services, Source BioScience, Nottingham, UK; Kamath et al., 2003).

**Immunostaining**

For embryo immunostaining, adult worms were placed into M9 on poly-l-lysine (0.01%)-coated slides and pressed under a coverslip to extrude embryos. For adult germline immunostaining, adult worms were placed in M9 buffer with 10 mM levamisole before worms were sliced open to extrude germlines. For both embryos and adult germlines, slides were laid on aluminum blocks pre-chilled by dry ice for more than 5 min. Coverslips were promptly removed to freeze-crack and permeabilize embryos and germlines. Both were then incubated overnight in methanol at −20°C. Prior to antibody treatment, fixed germlines were treated with 4% paraformaldehyde for one hour. Slides were blocked in 0.1% PBS Tween with 0.1% BSA for 1 hour, followed by an overnight incubation with 200 μL of KT3 primary antibody (1:100, DSHB). The following day, samples were incubated with a secondary antibody at room temperature for two hours (Jackson ImmunoResearch Labs Cat# 115-605-068 1:500). They were then mounted with Vectashield Antifade Mounting Media with DAPI.

**Spinning Disk Confocal imaging**

Imaging *C. elegans* embryos and germlines was carried out using a custom-built inverted Zeiss Axio Observer with CSU-W1 SoRa spinning disk scan head (Yokogawa), 4x relay lens (Yokogawa), fast piezo z-drive (Applied Scientific Instrumentation), and an iXon Life 888 EMCCD camera (Andor). Samples were illuminated with 405/637 nm solid-state laser (Coherent), using a 405/640 transmitting dichroic (Semrock) and a 624-40/692-40/525-30/445-45 nm bandpass filter (Semrock). Images were taken using Slidebook software using a 40x-1.3NA objective (Zeiss), with a 4x relay lens (Yokogawa) for embryos, and a 1x relay lens for germlines.

**Western Blot**

Recombinant proteins were diluted to 1 μM prior to denaturation and loading onto a 4–12% Bis-Tris pre-cast gels (Bio-Rad Hercules, CA). Western blot transfers onto PVDF membranes were run for 1 hour at 4°C. Membranes were blocked for 30 minutes in PBS with 5% milk. The KT3 antibody (DSHB) was diluted by 1:100 and incubated overnight. The secondary incubation occurred for two hours at room temperature (Thermofisher Scientific cat# 62-6720 1:4000, Jackson labs).

**PGL-1 and PGL-3 alignment**

The PGL-1 and PGL-3 C-termini were aligned by running amino acid sequences through MUSCLE (Edgar, 2004) , and then visualized via JALVIEW (Waterhouse et al. 2009) .
